# User Involvement in Research on Aging and Health: Creating Knowledge and Technologies With Older Adults

**DOI:** 10.1093/geroni/igaa057.2989

**Published:** 2020-12-16

**Authors:** Sofi Fristedt, Anna Wanka, Neil Charness

## Abstract

Although, user involvement is largely recognized as instrumental when developing relevant knowledge, services as well as products - aging populations are still likely to be sparsely involved in such processes. Surprisingly, many gerontechnologies are still developed based on a technological perspective rather than a gerontological perspective. Consequently, age-related changes as well as needs, actual use or perceptions of older adults are disregarded or neglected. Similar problems apply to public and private environments with potentially negative implications on accessibility. The present symposium includes four presentations that address user involvement, by capturing older adults’ and aging populations’ use as well as perceptions of emerging technologies, successful development of gerontechnologies, and a multigenerational mass-experiment on housing accessibility in later life. The first study from Germany captures the everyday situation of smartphone use as well as aspects of user experience, affect and social context among older adults. The second study addresses perceptions and attitudes of three generations in Sweden related to continuous technological advancement of products intended to support active and healthy aging. The third presentation will describe the iterative development process of the 2020 mass-experiment – the Housing Experiment -- involving older adults, stakeholders in the housing sector, teachers and pupils in Sweden. The fourth presentation from Canada explores the benefits, challenges, and solutions to support older adult engagement in research that leads to the successful development of technologies for and with older adults. Finally, our discussant will further elaborate on the respective study findings and summarize the symposium.

